# Long-Term Winter Population Trends of Corvids in Relation to Urbanization and Climate at Northern Latitudes

**DOI:** 10.3390/ani12141820

**Published:** 2022-07-17

**Authors:** Jukka Jokimäki, Marja-Liisa Kaisanlahti-Jokimäki, Jukka Suhonen

**Affiliations:** 1 Arctic Centre, University of Lapland, FI-96101 Rovaniemi, Finland; marja-liisa.kaisanlahti@ulapland.fi; 2 Department of Biology, University of Turku, FI-20014 Turku, Finland; juksuh@utu.fi

**Keywords:** birds, monitoring, cities, growth rate, crows, magpies, jackdaws

## Abstract

**Simple Summary:**

Corvids (e.g., crows, magpies and jays) are an important part of urban settlements, especially during winter. To understand the factors affecting the long-term population trends of corvids, we counted wintering corvids in 31 human settlements along a 920 km latitudinal gradient in Finland during four winters between 1991 and 2020. We detected a total of five corvid species, from which the Hooded Crow, the Eurasian Magpie and the Eurasian Jackdaw were found to be common. During the study period, the number of Eurasian Jackdaws increased, and their distribution range moved northwards. No corresponding changes were observed for the Hooded Crow or the Eurasian Magpie. Neither the local-level urban-, climate- nor food-related factors correlated with the changes in the numbers and growth rates of the corvids. No interspecific interactions were observed. We assume that the Eurasian Jackdaw has benefitted from the decreased persecution, and probably also from the large-scale climate warming. Our results suggest that urban settlements are quite stable wintering environments for generalist and omnivorous corvids.

**Abstract:**

Corvids (crows, magpies, jays) live in a close association with humans, and therefore knowledge about their population status and changes will be an essential part of monitoring the quality of urban environments. Wintering bird populations can track habitat and climate changes more rapidly than breeding populations. We conducted a long-term (1991–2020) winter census of corvid species in 31 human settlements along a 920 km latitudinal gradient in Finland. We observed a total of five corvid species: the Eurasian Magpie (occurring in 114 surveys out of 122; total abundance 990 ind.), the Hooded Crow (in 96 surveys; 666 ind.), the Eurasian Jackdaw (in 51 surveys; 808 ind.), the Eurasian Jay (in 5 surveys; 6 ind.) and the Rook (in 1 survey; 1 ind.). Only the numbers of the Eurasian Jackdaw differed between the study winters, being greater at the end of the study period (2019/2020) than during the earlier winters (1991/1992 and 1999/2000). The average growth rate (λ) of the Eurasian Jackdaw increased during the study period, whereas no changes were observed in the cases of the Hooded Crow or the Eurasian Magpie. The growth rate of the Eurasian Jackdaw was greater than that observed in the Finnish bird-monitoring work, probably because our data came only from the core area of each human settlement. Even though the number of buildings and their cover increased in the study plots, and the winter temperature differed between winters, the average growth rate (λ) of corvid species did not significantly correlate with these variables. These results suggest that urban settlements are stable wintering environments for the generalist corvids. The between-species interactions were all positive, but non-significant. Despite the total number of winter-feeding sites being greater during the winter of 1991/1992 than during the winter of 2019/2020, the changes in the numbers of feeding stations did not correlate with the growth rates of any corvid species. We assume that the Eurasian Jackdaw has benefitted from the decreased persecution, and probably also from large-scale climate warming that our study design was unable to take in to account. Our results indicated that wintering corvid populations succeed well in the human settlements in Finland. We recommend conducting long-term corvid research, also during breeding season, to understand more detailed causes of the population changes of corvids along an urban gradient. Without year-round long-term monitoring data, the conservation and management recommendations related to the corvid species in urban habitats may be misleading.

## 1. Introduction

Urbanization is one of the major causes of local, regional, and global biodiversity loss [1]. Urbanization changes the environmental structure and habitat compositions and causes a wide array of disturbances that correspondingly influence the living possibilities of species [2]. Some species suffer (urban avoiders), other species benefit (urban exploiters), whereas other species are quite independent (suburban adaptable species) from urbanization [3]. Typically, human-intolerant species are specialists, have restricted distribution ranges and are non-residents, whereas human-tolerant species are generalists (e.g., omnivores), widely distributed residents and behaviorally flexible species [4,5,6]. Some taxonomic groups can be remarkably successful at adapting to urban environments [2]. Many studies have indicated that especially sparrows *Passer* spp. [7], pigeons (e.g., Feral Pigeon, *Columba livia* domestica; [2]; Wood Pigeon *Columba palumbus*; [8]), and corvids (crows, magpies, jays, etc. [9,10,11,12,13,14,15]) benefit from urbanization all over the world. However, common species are often-neglected study objects despite their important ecological roles [16,17]. This is still true even after a heavy decrease in some common urban species, such as the House Sparrow (*Passer domesticus)* [7].

In the recent literature review, Benmazouz et al. [14] concluded that corvids have been successful in adapting to urbanized environments; >23% of the world’s 130 corvid species have been reported to live in cities. With their opportunistic way of life and plastic behavior, corvids are an excellent model group to study the impacts of urbanization on animals. Many corvid species thrive in many types of urban environments, from the peripheral urban areas to highly urbanized urban core areas [18,19,20,21]. Because of their wide distribution and good adaptability to many habitats, corvids are often described as urban exploiters or adaptors [13,22,23,24].

There are several possible reasons, that are not necessarily mutually exclusive, affecting the population trends of corvids in urban areas. For example, low levels of predation [25], good availability of (artificial) food resources [26,27,28,29] as well as an availability artificial nest sites [13,30,31,32,33] may benefit corvids. However, some studies have indicated that hunting activities [17,34] and persecution [12,14,35] may have negative influences on corvids. The ability to use novel anthropogenic resources such as food, safe nest and roost sites [36], as well as a high tolerance towards of conspecifics may allow some species, such as corvids, to thrive in urban environments [37]. Additionally, the recent climate warming may also especially influence the wintering birds [38,39,40,41,42,43].

Due to the overall success of corvids, the numbers of corvid–human conflicts might also increase [13,14,44,45,46]. For example, corvids have been reported to cause conflicts due to their aggressive behavior, droppings, disturbing noise as well as garbage scattering [14,45,46]. Therefore, it is especially important to monitor corvid numbers so that the conflicts can be avoided beforehand and possible management efforts can be targeted in the right way [46].

In some areas, such as in the United Kingdom [47] and Poland [48,49,50,51,52], special attention has been paid to the population trends of both rural and urban corvids. Currently, most of the earlier urban bird studies were conducted during the breeding season, whereas as fewer studies have examined urban birds during the winter [19,40,43,48,49,51,52,53,54,55,56,57,58,59,60,61,62,63,64]. However, because of the scarcity of resources and the severity of weather conditions in northern latitudes, winter is the most critical season for many birds [65,66]. Therefore, there is a special need for large-scale winter-season studies in the north [40,41], especially when wintering bird communities have been observed to track climate change faster than breeding communities [67].

It has been noted that urbanization buffers the seasonality of climate conditions and food availability [68]. For example, anthropogenic food resources provided either incidentally (e.g., garbage, leftovers) or intentionally (bird feeders) will increase both the food availability and predictability in urban habitats as compared to rural ones [62], thereby benefitting generalist corvid species such as the Eurasian Magpie (*Pica pica*) and the Hooded Crow (*Corvus corone cornix*) [29]. Additionally, milder microclimate (“urban heat island phenomena”) and less snow in cities than their surrounding rural areas might be beneficial for the urban wintering birds [42,65,69].

As most of the earlier urban bird studies have been very short, typically two years [2,70], there is an urgent need for long-term studies to evaluate in more detail the effects of urbanization on bird assemblages and populations [7,70]. Additionally, most of the earlier results of long-term winter bird studies are based on the works of voluntary birdwatchers. For example, Finland has a long-term tradition of monitoring wintering birds using volunteers since 1956 [71,72]. Based on these data, Fraixedas et al. [40] indicated that the numbers of wintering forest species have declined, whereas urban species have increased during 1959–2012 in Finland. They also highlighted that human-induced land-use changes were more important for the landbird species, whereas climatic factors were more important for the waterbirds. Moreover, Fraixedas et al. [40] suggested that urban species have benefited from the increase in the supplementary feeding of birds (see also [54,62,63]). Despite the important role of voluntary-based bird-monitoring work in general, biases due the differences in the observers’ skills to detect and identify birds over the years, the variable search efforts between winters, as well the changes in the sites sampled over the years will decrease the value of the voluntary-based data sets [73]. It may be possible that the population trends of corvid species might also differ from each other, partly due to the differences in their habitat needs, responses to climate change, or due to interspecific interactions.

The main aims of this study were (1) to analyze a long-term (1991–2021) corvid species winter-season composition, as well as their occurrence and abundance in Finland, (2) to evaluate the growth rate of different corvid species, and (3) to analyze the effects of multiple factors (latitude, urbanization, climate, food, interspecific interactions) on wintering corvids. We hypothesized that corvid species would differ in their responses to urbanization and climate change. We predicted that seed specialist corvid species, the (*Perisoreous infaustus*), the Nutcracker (*Nucifraga caryocatactes caryocatactes/macrohynchos*) and the Eurasian Jay (*Garrulus glandarius*), will avoid urban areas, whereas generalist species, such as the Eurasian Magpie, the Hooded Crow and Eurasian Jackdaw (*Corvus monedula*), will benefit from urbanization [74]. Earlier studies have indicated that climate warming with greater temperatures and less snow cover have increased the abundance of overwintering birds [38,42,43]. Therefore, we predicted that more corvids will nowadays overwinter in Finland, and we supposed that especially the southernly distributed, partially migrant species (the Eurasian Jackdaw) will benefit from climate warming. However, for the resident Eurasian Magpie, changes in the local habitat structure related to urbanization may be more important than climatic factors. Some earlier studies have indicated that larger corvid species dominate smaller ones (Hooded Crow > Eurasian Jackdaw> Eurasian Magpie > Eurasian Jay; [75,76]). As for whether the interspecific interactions would have any impacts on wintering corvid species, we predicted that there will be either negative or positive correlations between corvid species abundances and population growth rates.

## 2. Materials and Methods

### 2.1. Bird Surveys

We surveyed wintering corvids in the centers of 31 towns and villages (mean human population 21,694 ± 34,018 SD) along a 920 km south–north extent (60° N–68°; 950 km) in Finland for four winters (1991/1992, 1999/2000, 2009/2010 and 2019/2020). We established about 30 ha (mean 31.2 ha ± 7.7 SD) study plots within each study site. Each study plot was located at the most urbanized area of each study site. Twenty-nine study plots were surveyed during each winter, and the remaining two plots were surveyed during all winters except 2009–2010. All surveys were conducted during the mid-winter season (late December–early January) during good weather conditions (temperature not colder than −25 °C, not windy or rainy weather, no snowfall). All surveys were conducted during midday (between 10.00 and 15.00), i.e., when there is enough light to identify birds. 

We used the single-visit study plot method of Bibby et al. [77] to count wintering corvids. We walked through the study plot and also inspected the backyards of the buildings. This kind of survey method reduces many problems, e.g., varying visibility due to the buildings and noisy traffic, that are associated with counting birds in urban areas [60]. We used a relatively high survey speed (30 ha/h) to avoid possible double-counting of the same individual. We counted all the observed individuals, except the overflying ones that did not land and stay on the study plot. Most of the surveys (78% out of all 122 surveys) were conducted by two professional ornithologists (JJ; JS) with over tens of years of experience in conducting winter bird surveys. Additionally, the other surveyors had much experience with this kind of study. Therefore, the possible inter-observer bias is minimal in our study.

Because of practical reasons, we used the single-visit survey method to obtain a reasonable number of spatial replicates for statistical analysis. The short mid-winter days (4 h) at northern latitudes, the long distances between the study sites, and the unfavorable weather conditions during the survey periods decreased the possibility of conducting multi-visit surveys without losing spatial replicates. We are familiar with the possible problems associated with single-visit surveys. Our earlier winter bird studies conducted in Finland have indicated that a single-visit survey in urban settlements will detect about 90% of the species and 80% of the individuals [59,60]. Therefore, we suppose that the efficiency and accuracy of the winter-season bird surveys are high. It should also be noted that a scarce vegetation cover during the winter, due to the absence of leaves on deciduous trees and shrubs, increases the visibility of birds, and thereby the survey efficiency. However, we further estimated the efficiency of the single-visit census by repeating the surveys five times during the winter of 2019/2020 in six study plots. Half of them were located in southern (60° N) and half in northern Finland (66° N). In both southern and northern Finland, one site was located in a large city (Turku (195,301 inh.) and Rovaniemi (64,194 inh.)), one in a medium-sized city (Naantali (19,579 inh.) and Kemijärvi (7107 inh.)) and one in a village (Aura (3958 inh.) and Muurola (891 inh.)).

### 2.2. Explanatory Variables

We extracted the average number of inhabitants, the average area of buildings and the number of buildings from each study plot and each study year from the 250 m × 250 m square database (© SYKE and TK, 1990, 2000, 2009 and 2019). The grids used covered the whole study plot (see more details; [7]). 

We extracted the average daily winter months’ (December–February) temperature (°C), the arrival date of the permanent snow cover (a continuous snow cover with at least 5 cm snow that stayed on the ground for at least 7 days), and the amount of snow (cm) on the 15th of December from nearest meteorological station of each study plot by using the Finnish Meteorological Institute database (see more details; [7]). We counted the number of active feeding sites (i.e., sites that contained food during the surveys) within the study plots during the first and last bird-survey winters (1991/1992 and 2019/2020) to estimate the change in artificial food availability.

Based on our earlier analyses related to the changes in explanatory variables [7], the number of buildings and their cover increased during the study period; the total number of buildings was lower during the first study winter than during the later ones, and the built-up cover of the first and second study winters were lower than during the later study winters. However, the number of inhabitants in the study plots did not differ between the study winters.

According to Jokimäki et al. [7], the winter temperature differed between all study winters. The coldest winter was 2009/2010 followed by 1999/2000, 1991/1992 and 2019/2020. The amount of snow did not differ between the study winters, but the snow cover arrived earlier during 1991/1992 than the other winters.

The change in the number of feeding sites (delta number of feeding sites) did not correlate significantly (*p* > 0.05) with the number of buildings, the changes in the area of buildings, or the number of inhabitants in the study plot [7]. However, more winter-feeding sites were detected during the beginning of the study (1991/1992) than at the end of the study period (2019/2020; [7]). During the surveys, avian predator species (Eurasian Sparrowhawk, *Accipiter nisus*; Northern Goshawk, *Accipiter gentilis*) were detected only during nine surveys out of 122, and no cats were observed in any surveys [7]. Therefore, we suppose that the possible influence of predators on corvids has been minimal in our study.

### 2.3. Statistical Methods 

Because of the low occurrence level and low abundances of the Eurasian Jay (5 records with 6 individuals) and the Rook (1 record with 1 individual in the city of Oulu during 1991/1992), these species were excluded from further analyses. We estimated population growth rates (λ) of the corvid species with time-interval-corrected methods (Morris [78]: λ = log10 (N_i+1_/N*_i_*)/(study interval), where study interval = √(t_i+1_ − t_i_), t_i_ = year of study winter; and N_i_ = number of individuals during study winter i and N_i+1_ = number of individuals during study winter_i+1_). If we did not find any corvids either during a previous study winter or a later study winter within the same study area (i.e., in cases when N_i+1_ = 0 or N_i_ = 0), we added one ’unseen individual’ for the data set; otherwise, it is impossible to calculate λ-value. Therefore, the method of calculating λ-values is conservative. We used an average λ-value of each study area as an independent observation for statistical tests. We used the Friedman’s two-way ANOVA to test differences in the Eurasian Jackdaw, Hooded Crow and Eurasian Magpie abundances between the four study winters. The study site was used as a “block” variable in the Related-Samples Friedman’s Two-Way Test. In this analysis, we excluded two study sites (Ruokolahti and Muhos), which only had data from three winters. The Related-Samples Friedman’s Two-Way Test was also used to study differences in site-specific urban factors as well as climatic factors between the study winters. After checking that the assumptions of the parametric test were fulfilled, we used the Pearson correlation to examine the relationship between the population growth rate (λ) of different corvid species and the changes (delta) in the number of inhabitants, average area of buildings and number of buildings within the study plot as well as along the latitudinal gradient. Differences in the environmental variables between the last and the first study year was used as a delta value. Latitude was measured as kilometers from the most southern study area (=0 km) to the most northern one (=920 km). We used the Spearman Rank Correlation to examine the relationship between the population of growth rate (λ) of the corvid species and the changes (delta) in the number of feeding sites between the 1990/1991 and 2019/2020 because the data did not fulfill the assumption of the parametric statistical test.

We used the coefficient of variation (CV% = 100 × (SD/mean)) to estimate the variability in the abundance of corvid species within a single winter. The greater the value, the more variable are the results of different surveys. The CV% value can be used as a conservative estimate of the efficiency of the single-visit survey.

All the data analyses were performed using the IBM SPSS statistical package, version 26.

## 3. Results

### 3.1. Within Winter Variation of Corvid Populations

The coefficients of variation (CV%) of the abundance did not differ between the corvid species (Eurasian Jackdaw 100 ± 70 (mean ± SD; n = 6); Hooded Crow 60 ± 50; Eurasian Magpie 60 ± 30; F_2,14_ = 1.151, *p* = 0.344) in our five-visit surveys during a single winter. The latitude did not have any influence on the CV% (*t*-tests; all *p* > 0.05). Urbanization influenced only the CV% of the Hooded Crow (F_2,3_ = 10.300, *p* = 0.045), with the CV% being greater in the small-sized settlements (mean ± SD; 114 ± 32) than in the mid-sized settlements (18 ± 6; Tukey HSD post-hoc pairwise comparison; *p* = 0.042).

### 3.2. Corvid Populations

A total of five corvid species were observed during the surveys: the Eurasian Jackdaw, the Hooded Crow, the Eurasian Magpie, the Eurasian Jay, and the Rook (Table 1). 

**Figure 1:**
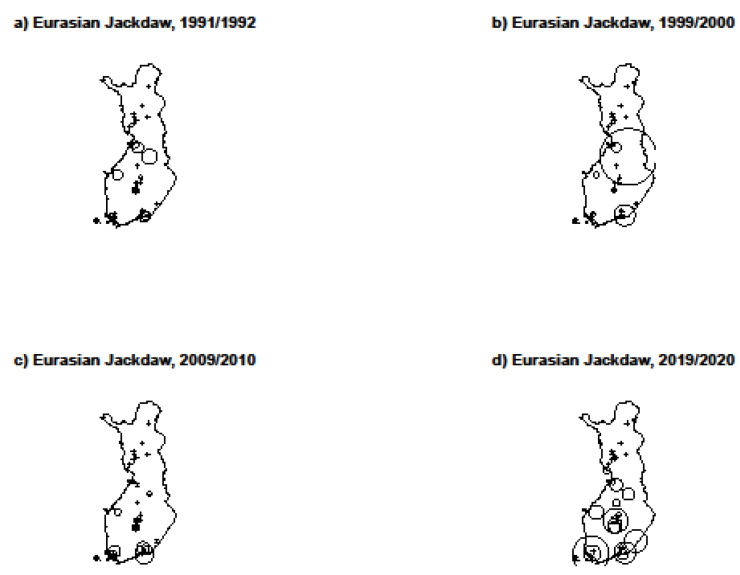
Distribution maps of Eurasian Jackdaw populations in 31 study sites during the winters of 1991/1992 (**a**), 1999/2000 (**b**), 2009/2010 (**c**) and 2019/2020 (**d**) in Finland. The size of the circle indicates the number of individuals. The smallest circle indicates one individual, and the largest circle 111 individuals. The symbol (+) indicates lack of observation during a specific winter within a specific study site.

The number of Eurasian Jackdaws differed between the study winters (Related-Samples Friedman’s Two-Way Test, χ^2^ = 17.95, df = 3, *p* < 0.001; Table 1; Figure 1). In the pairwise comparisons, more Eurasian Jackdaws were observed during the winter of 2019/2020 than 1999/2000 (Related-Samples Friedman’s Two-Way Test; χ^2^ = −1.02, *p* = 0.003) and 1991/1992 (Related-Samples Friedman’s Two-Way Test; χ^2^= −0.81, *p* = 0.017). The numbers of the Hooded Crow(Related-Samples Friedman’s Two-Way Test, χ^2^ = 7.39, df = 3, *p* = 0.060) and Eurasian Magpie (Related-Samples Friedman’s Two-Way Test; χ^2^ = 2.49, df = 3, *p* = 0.77) did not differ between the study winters (Table 1).

**Figure 2:**
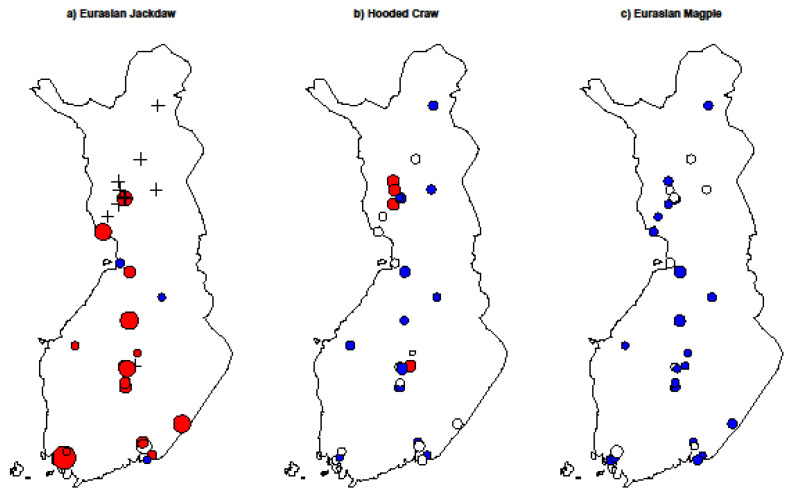
Population growth rates (λ) of the Eurasian Jackdaw (**a**), Hooded Crow (**b**) and Eurasian Magpie (**c**) populations in 31 study sites during 1991/1992–2019/2020 in Finland. The size of the dot indicates the population growth rate (the larger the dot, the greater the change) and the color of the dot indicates direction of the growth (red positive; blue negative; and open dot zero growth rates). The symbol (+) indicates a lack of observation during the four winters.

The average growth rate (λ) of the Eurasian Jackdaw (Figure 2a) increased during the study winters, whereas no changes were observed in the Hooded Crow (Figure 2b) or the Eurasian Magpie (Figure 2c; Table 2).

### 3.3. Effects of Latitude, Urbanization, Number of Feeding Sites and Weather Conditions

The average growth rate (λ) of the corvid species did not significantly correlate with the latitude, average area of the buildings, number of buildings and number of inhabitants in the study plot (Table S1) or any weather conditions (Pearson correlations; *p* > 0.05 in all cases; results not shown).

The correlation coefficients between the changes in the average growth rate of species (λ) and changes (delta) in building area, number of buildings and number of inhabitants and number feeding sites in the study plots (Table S2) or all weather variables (Pearson r; *p* > 0.05 in all cases; results not shown) were all non-significant. Changes in the numbers of feeding stations between the 1991/1992 and 2019/2020 did not correlate with the growth rates of any corvid species (Spearman´s rho; all cases *p* > 0.05; n = 31, except in the case of the Eurasian Jackdaw n = 21). The between-species correlations in growth rates were all positive, but non-significant (Eurasian Magpie vs. Hooded Crow (Pearson correlation), r = 0.303, *p* = 0.097, n = 31; Eurasian Magpie vs. Eurasian Jackdaw, r = 0.384, *p* = 0.086, n = 21; and Eurasian Jackdaw vs. Hooded Crow, r = 0.201, *p* = 0.382, n = 21).

## 4. Discussion

### 4.1. Within Winter Variation of Corvid Populations 

The coefficients of variation of the abundances (CV%) within a single winter did not differ between the corvid species, and the latitude did not influence the CV% values of any corvid species. These results indicate that the results of single-visit surveys are comparable between species, and single-visit surveys provide a reliable estimate of the abundance of corvid species during the winter (See also [57,60]). Urbanization level had only a minor influence on the CV% values of the corvids; the only significant result was that the CV% of the Hooded Crow was greater in the small- than in mid-sized settlements. These results agree well with the results of Jokimäki and Kaisanlahti-Jokimäki [57], who noted that the CV% of the Hooded Crow was greater in less urbanized villages than in residential areas of cities in northern Finland. This indirectly indicates that urban habitats are better wintering habitats for crows than rural ones in northern Finland.

### 4.2. Wintering Corvid Species Composition

Our results indicated that fewer corvid species are urbanized in Finland than in southern and central Europe. For example, we detected the Rook only once even though the species is very abundant in more southern urban areas (e.g., [48,49,79]). The Rook became a member of the Finnish avifauna no earlier than the late 1800s, and it is still breeding in only a few sites in Finland [80]. Additionally, the Rooks migrate to central and southern Europe during the late autumn from Finland [81]. Therefore, it was not a surprise that the species was rare in our winter-season samples.

The Raven is urbanized in some European countries, such as Ukraine [19] and Poland [82], but in Finland, as well as in many other parts in Europe, the Raven is a typical species that avoids human-caused disturbances partly due to the long-term persecution [80,83]. For example, the main cause of death of the Finnish ring-marked Ravens has been deliberate killing (88%; n = 1043, [81]). Since 1993, the Raven has been protected in Finland, except in the reindeer-herding area (=northern Finland) where the species is still unprotected outside the breeding season (10.4–31.7). However, there are some observations that a few individuals can nowadays also be detected in urban areas [80,81], such as in Rovaniemi (personal observations; JJ). The decreases in persecution pressure will probably eventually lead to the colonization of the Raven in suburban areas of Finnish cities in the future. However, Ravens hold territory during the entire year in the vicinity of their nesting locality and will occasionally go for a “winter feeding trip”, e.g., in rubbish dumps. Therefore, it is possible that the closing of rubbish dumps influences the winter distribution of the Ravens. In addition, it might be possible that there is a lack of suitable nesting sites, e.g., high trees, high-voltage electric poles, or abandoned solitary buildings [14], in urban areas for the Ravens in Finland.

Despite that the Eurasian Jay has urbanized in central and southern Europe (e.g., [79,84,85,86,87]), we detected jays only in five surveys. Additionally, Jokimäki and Kaisanlahti-Jokimäki [58] indicated in their local-level winter study in Rovaniemi, Finland, that Eurasian Jays avoid urban areas. Moreover, the results of Matsyura and Jankowski [19] from the city of Zhytomyr (Ukraine) indicated that Eurasian Jays prefer less-urbanized suburban areas over more urbanized ones. The Eurasian Jay has obviously benefitted from the climate warming since its population range has expanded in many European countries [87,88], and also in Finland [80]. The Eurasian Jay is also nowadays more common and abundant in the Finnish winter-feeding sites, especially in northern Finland [29]. It might be possible that winter feeding will help the urbanization process of the Eurasian Jay by decreasing the migratory needs of the species, as has happened in the cases of the Mallard (*Anas platyrhynchos*; [89]) and the Eurasian Blackbird (*Turdus merula*, [90]). Additionally, our winter data show some range expansion towards the north, although our data are not large enough for detailed analyses.

The two small-sized corvids, the Nutcracker (the races *Nucifraga caryocatactes caryocatactes* and *N. c. macrohynchos*) and the Siberian Jay (*Perisoreous infaustus*), were not observed in our study sites even though both species occurred in the near surroundings of some study sites. The Nutcracker race *macrohynchos* is an invasive species that sometimes invades from Siberia to Finland, and these individuals have established breeding populations in the periurban areas of some Finnish cities such as the study cities Rovaniemi and Jyväskylä (personal observations; JJ & JS). The Nutcracker race *caryocatactes* inhabits only the southwestern parts of Finland and could also be observed in our study cities Raisio and Naantali (personal observations; JS). Both Nutcracker species are seed-eating specialists; the *macrohynchos* race uses the seeds of the Siberian pine seeds (*Pinus cembra* subspecies *sibirica*), whereas the race *caryocatactes* uses the seeds of the common hazel (*Corylus avellana).* Even though both the Nutcracker and the northerly distributed Siberian Jay often use feeding sites during winter [29], both species avoid highly urbanized areas. Planting sembras and common hazels will obviously help the urbanization process of the Nutcracker in the future. Additionally, increased winter feeding by peanuts has benefitted the Nutcracker in Finland [29]. Because the Siberian Jay is a very sedentary species that prefers continuous coniferous old-growth forests [80], it is unlikely that the species will colonize scare, fragmented young-aged and deciduous-dominated urban green areas, even in the future.

According to our results, the Eurasian Magpie, the Hooded Crow, and the Eurasian Jackdaw were the most common and abundant wintering corvid species in human settlements in Finland as detected in the Finnish winter bird-monitoring studies (see also [29,91]. As compared to the rare, seed-specialist corvid species in Finland, all of these abundant corvid species are generalists. The distribution range of the Eurasian Jackdaw has been restricted in southeastern parts of Finland until 1950s; thereafter, the species has increased in numbers and its distribution range expanded toward the north during 1984–2010 [80], and especially during 2010–2020 [72].

### 4.3. Changes in Occurrences, Abundances and Growth Rates of Corvids

Our results indicated that the number of Eurasian Jackdaws has increased, and their winter distribution range has expanded northwards during the last 30 years. These results agree well with the results of the voluntary-based Finnish winter bird-monitoring work [40,72], indicating the usefulness of voluntary-based winter bird-monitoring work. The average growth rate (λ) of the Eurasian Jackdaw has also increased during the study winters. However, our data indicated a greater increase rate of the Eurasian Jackdaw than the Finnish bird-monitoring work ([40]; Table S3). One reason for this difference is that the data of the Finnish winter bird census work covers all kinds of habitats, whereas our data are restricted only to the most urbanized parts of cities and villages. Obviously, the Eurasian Jackdaw has succeeded better within urban settlements than in rural areas in Finland.

According to our results, the first increase in the wintering Eurasian Jackdaws started during the 1990s, and the second one during the late 2010s. These records correspond well with the results of Lehikoinen and Tirri [72] who indicated that the number of wintering Eurasian Jackdaws heavily increased from 1980–1999 to 2010–2020 in Finland. At the same time as the increase in numbers, the wintering range of the Eurasian Jackdaw also moved northwards (this study; [72]). However, according to the results of European breeding bird atlas work, there are differences in Eurasian Jackdaw breeding distribution changes and populations trends (1974–1989 vs. 2013–2017) between European countries [92]. For example, the Eurasian Jackdaw population has been relatively stable during the last 30 years in Warsaw, Poland [49].

We did not find any significant range changes of other corvid species than the Eurasian Jackdaw during the winters of 1991/1992–2019/2020 in Finland. These results are mainly consistent with the results of national European and Finnish breeding-bird-atlas-monitoring work. According to the European bird-atlas-monitoring work, the distribution ranges of the crows and magpies have been quite stable during the years 1972–2017 [93,94]. Additionally, the overall European population trend of the crows has been stable [93], whereas the magpie numbers firstly increased in the 1970s and 1980, and thereafter, depending on the country, either remained stable, decreased, or increased [94]. These differences in population trends between countries suggest that in addition to large-scale factors (such as climate), local/regional-scale factors also influence the population trends of corvids.

According to the Finnish breeding-bird-atlas-monitoring work (1974–1979–2006–2010), the distribution range of the Eurasian Jay moved towards to the north during the early 2000s, whereas the distribution range of the Eurasian Magpie and the Hooded Crow has not changed [80]. According to the Finnish winter bird census data (1967–1969; 1980–1999; 2010–2020), there have been some changes in the center of gravity of density in overwintering corvid species [72]. The center of gravity of the Hooded Crow has moved 277 km northwards, and the Eurasian Magpie 125 km and the Eurasian Jackdaw 33 km, correspondingly [72]. The results of Väisänen [29] show that more Eurasian Jays and Hooded Crows overwinter nowadays in northern Finland, and these individuals often use feeding sites. According to the voluntary-based Finnish winter-feeding-site-monitoring work 1991–2020, the Eurasian Jay has become more common and abundant, and the Eurasian Magpies and Hooded Crows occurrence and numbers have increased in northern Finland, but not in southern Finland [29]. In general, the results of the Finnish winter bird-monitoring work indicate that more birds, also corvids, overwinter nowadays in Finland [29,72].

### 4.4. Causes of Population Changes of the Three Most Abundant Corvid Species

There are several possible reasons, that are not necessarily mutually exclusive, for the population changes of corvids in Finland and Europe. In addition, these factors may operate hierarchically. For example, climate change may influence habitat structure, which in turn can influence food availability, and changes in food availability may influence interspecific interactions. In the following section, we discuss the roles of these factors by applying the IUCN Threat Classification Scheme [95] and by recording the timing (past, ongoing, future) of the drivers.

#### 4.4.1. Climate Change

There are several possible reasons for the population changes of corvids in Finland. The Eurasian Jackdaw is a partial migrant species in Finland, and the ongoing climate warming might have been advantageous for the Eurasian Jackdaw. During our study period (1991–2020) the average winter-season temperature increased 1–2 °C in Finland [29]. Correspondingly, we observed that the abundance of the Eurasian Jackdaw was higher at the end of the study period (2019/2020) than during the earlier study winters (1991/1992 and 1999/2000), supporting the climate-warming hypothesis to some extent. A warmer climate means less snow cover, and this will be advantageous for the ground-foraging species such as the Eurasian Jackdaw [42,43,52]. Additionally, Virkkala and Lehikoinen [96] have indicated a climate-based breeding-season-range expansion of the Eurasian Jackdaw since the mid-1970s in Finland. The importance of this driver will obviously increase in the future. For example, fewer Finnish Eurasian Jackdaws will migrate to central Europe, and this can lead to a decrease in wintering Eurasian Jackdaws in central Europe.

#### 4.4.2. Persecution and Hunting 

Both the Eurasian Magpie and the Hooded Crow have been heavily persecuted (e.g., nests destroyed) and large numbers of adults were deliberately killed until the late 1960s and early 1970s in Finland [80,81]. After the persecution decreased, magpies started to colonize even the most urbanized core city areas during the 1980s in Finland [23]. According to Vuorisalo et al. [12], Finnish crows have benefitted from the decreased persecution in cities since the 1960s. This has led to the habituation of crows towards humans, and even their expansion into the core areas of cities [12]. In Finland, the main cause of death of the Finnish ring-marked Eurasian Jackdaws has been deliberate killing (39%; n = 647, [81]). However, the proportion of the killed Eurasian Jackdaws from all dead recoveries has decreased from 90% to under 10% during the last five decades in Finland [81]. According to Antikainen [97], the protection status of the Eurasian Jackdaw has varied a lot in Finland, and this has obviously influenced the population dynamics of the Eurasian Jackdaw. Before the year 1993, the Eurasian Jackdaw was unprotected as it was considered a harmful pest species. Later, during the year 1993, the Eurasian Jackdaw was protected due to the population decline, but in August 2018 the species was again allowed to be hunted due to their population increase. During the spring of 2019, the Eurasian Jackdaw received a breeding time protection (10.3–3.7) in Finland. Our data indicate that the number of Eurasian Jackdaws increased between 1991 and 1999 as well as between 2010 and 2019. Therefore, it is possible that the full protection during the year 1993 and the partial breeding-season protection during the spring of 2019 has benefitted the Eurasian Jackdaw. The decreased persecution of the species in cities during the last few decades, as opposed to rural areas, appears to be an important factor promoting the corvids’ tolerance and habituation to urban environments [12,23,98,99,100].

#### 4.4.3. Food Factor

One possible reason influencing the abundances and trends of corvids is the food availability [101]. Szala et al. [32] detected a positive correlation between the winter abundance of the Eurasian Jackdaw and the number of garbage cans in the city of Poznań, Poland. However, Vuorisalo et al. [12] suggested that the local overabundance of food does not fully explain the sudden increase in the Hooded Crow population density in the 1960s in Finland. Despite the fact that the total number of winter-feeding sites has decreased from 1991/92 to 2019/2020, we did not find any significant correlations between the growth rates of the corvid species and the changing number of feeding sites. These results partly differ from Väisänen [29] who has indicated that some species, such as the Eurasian Jay and the Hooded Crow, have benefitted from the intentional winter feeding of birds in Finland. Similar results to Väisänen [29] have also been detected in Britain, where the Eurasian Magpie, the Eurasian Jackdaw, the Carrion Crow (*Corvus corone* corone) and the Eurasian Jay increased their garden-feeder use during 1973–2012 [97]. However, the recent decline in the Eurasian Jackdaw populations in several European cities has been related to the lack of good-quality food [102,103].

Many corvid species use rubbish dumps (landfill sites) as foraging areas (see, e.g., [76]). However, partly due to the new EU waste legislation, Finland also renovated the waste law so that the waste collection was centralized and the number of rubbish dumps was heavily decreased (Waste law 1072/1993; [104]. For example, the number of rubbish dumps has been decreased from over 550 sites in 1990 to about 75 sites in 2010 in Finland [103]. Additionally, since the year 2016, it was not allowed to transport biowastes, which are important food sources for corvids, to the rubbish dumps in Finland [104]. We assume that the changed waste legislation has influenced corvids´ food availability and foraging areas, but unfortunately, detailed scientific studies related to the changed legislation and the corvids are still lacking. In our case, the increase in the Eurasian Jackdaw numbers happened during 1991–1999 and 2010–2019, i.e., just when the new waste legislation was implemented (1993) and when biowaste transportation to the rubbish dumps was ended (2016) in Finland. It is possible that the reduction in rubbish dumps, the increased distance between the individual rubbish dumps and cities, as well as the decrease in biowastes in rubbish dumps have forced, e.g., the Eurasian Jackdaws, to seek food within urban areas more often.

#### 4.4.4. Interspecific Interactions

Interspecific interactions may influence the abundance of corvid species [75,76]. Our results indicate that the between-species correlations in growth rates were all positive, but non-significant, suggesting that some factors affect different corvid species in a similar way, and that there is no interspecific competition between corvid species during the winter season in Finland. Additionally, Szala et al. [32] and Zmihorski et al. [52] have found positive associations between wintering corvid species in Poland. In addition, Waite [105,106] has observed that the overlap of the winter-season habitat use of four sympatric corvid species is large [105] and that the overlap of the resource use of corvids is insufficient to support the occurrence of interspecific aggression [106]. However, there are also observations that crows may chase-attack other corvids [76,106]. More experimental studies are needed to understand in more detail the possible role of interspecific competition on the habitat use of corvids, both during the winter and the breeding seasons.

#### 4.4.5. Other Factors

Sorace and Gustin [107] have indicated that the level of urbanization may influence the distribution and abundance of corvids. However, even though the urbanization level of our study sites increased during the study period, it did not have any influence on corvid numbers or their population growth rates. There are two alternative reasons for these conflicting results. Firstly, our study was conducted during the winter season, whereas Sorace and Gustin´s [107] study was conducted during the breeding season. It is well-known that birds are more opportunistic in terms of their food choice and habitat use during the winter than any other season (e.g., [65], and therefore the impact of urbanization level may have a minor effect on the wintering corvids. Secondly, Sorace and Gustin´s [107] study was conducted in southern latitudes in Italy, whereas our study was conducted in northern latitudes. It is possible that the habitat needs of southern and northern corvid populations may differ. We did not detect any direct influence of the changing climate on the abundance or growth rates of corvids. However, we suppose that the range expansion of the Eurasian Jackdaw towards to the north can be at least partly due to the climate warming during 1990–2020 in Finland (see e.g., [42]).

As in all ecological studies, our study also has weaknesses. It might be possible that our habitat-level variables are too general to detect detailed information about the habitat needs of corvids. For example, according to the results of the winter corvid study conducted in Poland, Hooded Crows benefitted from the increased park area, the Eurasian Jackdaw was positively affected by the number of garbage cans, and Eurasian Magpie numbers were positively associated with the number of walnut trees and garbage cans within the study plot [79]. We evaluated habitat-related changes within a very small scale (about 30 ha) and only from the most urbanized areas of each study site (in which the predicted changes are assumed to be smallest). Therefore, we excluded habitat factors operating at the larger landscape scale. We used discreate meteorological data from our four study winters instead of using continuous data like Fraixedas et al. [40] and Lehikoinen and Tirri [72]. Due to these spatial- and temporal-scale-related topics, it might be possible that we were unable to detect the possible importance of the urbanization and climatic factors for the changes in wintering corvid populations. For example, we assume that the long-term (1961–2014) decreases in snow-cover length and depth, especially in southern Finland, ref. [108] have been beneficial for the ground-foraging corvids, such as the Eurasian Jackdaw.

## 5. Conclusions

The wintering corvid species composition in urban settlements in Finland is less diverse than in central or southern Europe, partly due to fact that not all corvid species, such as the Eurasian Jay, have urbanized in Finland yet. Additionally, we suppose that harsh northern winter conditions restrict the wintering occurrence of short- and partially migratory species, such as the Rook, to overwinter abundantly in Finland. However, there are some remarks that due to the recent climate warming, more Eurasian Jays, Hooded Crows and Eurasian Jackdaws overwinter nowadays in Finland. All of these species are either short-distance or partial migrants, and milder climates with less snow means a shorter migration or even a residential way of life in the near future. From the three common and abundant overwintering corvid species (Hooded Crow, Eurasian Magpie and Eurasian Jackdaw), only the partial migratory jackdaw numbers, distribution range and growth rate increased significantly during the recent decades in Finland. Our results suggest that urban settlements are quite stable wintering environments for the omnivorous corvids. We did not detect any significant interspecific correlations between species, and therefore we suppose that there is no great between-species competition for resources within urban settlements during the winter season. However, our study was conducted during the winter seasons when the habitat use of birds is supposed to be more opportunistic or plastic than during the breeding season. Therefore, our results cannot be generalized for a season other than winter, nor are they directly transformable for the more southern areas. Additionally, our study is a descriptive one, and more experimental studies about factors affecting the winter mortality and breeding reproductivity of corvids are urgently needed. We recommended conducting more long-term corvid research during both the winter and breeding seasons to understand in more detail the population trends of corvids, in order to be able to manage urban corvid populations when needed. Additionally, we suggest applying the IUCN threat analysis approach in more detail for the corvids, e.g., by evaluating the scope (i.e., how large a proportion of the population different drivers are affecting) and the severity of different drivers.

## Figures and Tables

**Table 1:** Mean abundances, standard deviations, and minimum and maximum numbers of detected individuals by species during different study winters within study plots.

Species	Mean	SD	Min	Max	N
Eurasian Jackdaw					
Winter 1991/1992	3.7	6.6	0	25	31
Winter 1999/2000	6.0	20.7	0	111	31
Winter 2009/2010	3.8	7.5	0	32	29
Winter 2019/2020	12.8	16.6	0	69	31
Hooded Crow					
Winter 1991/1992	5.2	7.2	0	31	31
Winter 1999/2000	5.0	8.7	0	45	31
Winter 2009/2010	9.0	10.4	0	41	29
Winter 2019/2020	4.7	4.9	0	24	31
Eurasian Magpie					
Winter 1991/1992	7.6		6.3	0	28
Winter 1999/2000	8.5	5.7	0	19	31
Winter 2009/2010	8.2	5.6	1	26	29
Winter 2019/2020	8.2	5.7	0	25	31

**Table 2:** Changes in the average growth rate (λ) of the three corvid species during 1991–2020 in Finland. Test of lambda against the zero-growth rate (one-sample *t*-test and test value = 0) in three wintering corvid species in Finland.

Species (N)	Mean	SD	t	*p*
Eurasian Jackdaw (21)	0.2854	0.3039	4.305	<0.001
Hooded Crow (31)	0.0076	0.1218	0.346	0.732
Eurasian Magpie (31)	0.0296	0.1242	1.325	0.195

## Data Availability

Data will be available from the authosr by request.

## Supplementary Materials

The following supporting information can be downloaded at: https://www.mdpi.com/article/10.3390/ani12141820/s1. Table S1: Correlation coefficients (Pearson r) between the growth rate (λ) of the corvid species and latitude, average building area, average number of buildings and average number of inhabitants in the study plots. Table S2: Correlation coefficients between the average growth rate (λ) of species and changes (delta) in building area, number of buildings and number of inhabitants and number feeding sites in the study plots. Table S3: Differences in growth rates (λ) of corvid species between the current data and Finnish bird monitoring data (the One Sample *t*-test).
