# Supplementary material for: Long-Term Winter Population Trends of Corvids in Relation to Urbanization and Climate at Northern Latitudes
Source: Animals (Basel). 2022 Jul 17;12(14):1820. doi: 10.3390/ani12141820 (PMC9311560; doi:10.3390/ani12141820)
Supplement: Supplementary file 1 [file animals-12-01820-s001.zip › animals-1796297-supplementary.pdf]

Jokimäki et al. Supplementary tables: Table S1-S3.

Table S1. Correlation coefficients (Pearson r) between the growth rate (λ) of the corvid species and latitude, average building area, average number of buildings and average number of inhabitants in the study plots. Sample sizes are n = 31, except in the case of the Eurasian Jackdaw, n = 21.

	Latitude	Building area	Numb. of buildings	Numb. of inhabitants
Eurasian Jackdaw	0.266	-0.166	-0.234	0.061
	$p = 0.696$	$p = 0.472$	$p = 0.307$	$p = 0.793$
Hooded Crow	0.266	-0.145	-0.066	-0.125
	$p = 0.149$	$p = 0.435$	$p = 0.725$	$p = 0.501$
Eurasian Magpie	-0.075	0.089	0.142	0.128
	$p = 0.685$	$p = 0.603$	$p = 0.448$	$p = 0.492$

Table S2. Correlation coefficients between the average growth rate (λ) of species and changes (delta) in building area, number of buildings and number of inhabitants and number feeding sites in the study plots. Differences in the environmental variables between the last and the first study year was used as a delta value. Sample sizes are n = 31, except in the case of the Eurasian Jackdaw, n = 21.

	Building area	Numb. of buildings	Numb. of inhabitants	Numb. of feeding sites
Eurasian Jackdaw	0.195	0.329	-0.018	-0.301
	$p = 0.398$	$p = 0.146$	$p = 0.938$	$p = 0.851$
Hooded Crow	0.143	0.082	0.125	-0.091
	$p = 0.442$	$p = 0.666$	$p = 0.502$	$p = 0.627$
Eurasian Magpie	-0.214	-0.099	0.028	-0.310
	$p = 0.248$	$p = 0.598$	$p = 0.882$	$p = 0.090$

Table S3. Differences in growth rates (λ) of corvid species between the current data and Finnish bird monitoring data (the One Sample t-test). Growth rate values of the current study and the Finnish bird monitoring data (obtained from the Appendix 1 of Fraixedas et al. [40]) are given in parentheses.

Species (Lamda values)	t	df	*p*
Eurasian Jackdaw (0.2854/0.0028)	4.262	20	<0.001
Hooded Crow (0.0076/-0.0060)	0.620	30	0.540
Eurasian Magpie (0.0296/0.0104)	0.859	30	0.397
